# Comparative Gastrointestinal Digestion Dynamics of Air-Dried and Freeze-Dried Yak Jerky: Insights from a Dynamic *In Vitro* Human Stomach–Intestine (DHSI-IV) System

**DOI:** 10.3390/foods14122086

**Published:** 2025-06-13

**Authors:** Bei Xue, Zhendong Liu, Yiling Wen, Yubing Lu, Yidan Zhang, Jingjing Wang, Xiao Dong Chen, Peng Wu

**Affiliations:** 1 Clinical Medical Research Center for Plateau Gastroenterological Disease of Xizang Autonomous Region, and School of Medicine, Xizang Minzu University, Xianyang 712082, China; bxue@xzmu.edu.cn (B.X.); lyb202309@126.com (Y.L.); 17792825747@163.com (Y.Z.); 2 Food Science College, Xizang Agriculture and Animal Husbandry University, Nyingchi 860000, China; liu304418091@126.com (Z.L.); 18213577130@163.com (Y.W.); 3 Life Quality Engineering Interest Group, School of Chemical and Environmental Engineering, College of Chemistry, Chemical Engineering and Materials Science, Soochow University, Suzhou 215123, China; xdchen@mail.suda.edu.cn

**Keywords:** yak jerky, *in vitro* gastrointestinal system, proteins, air drying, vacuum freeze drying, gastric emptying

## Abstract

Yak meat jerky, a traditional high-protein food commonly consumed in high-altitude regions, is often produced via air-drying, which may adversely affect its nutritional quality and digestibility. This study systematically compared the gastrointestinal digestion profiles of air-dried yak meat (ADM) and vacuum freeze-dried yak meat (VFDM) using a dynamic *in vitro* human stomach–intestine (DHSI-IV) system. Key digestive parameters, including gastric emptying kinetics, particle size distribution, and protein hydrolysis, were evaluated under physiologically relevant conditions. VFDM exhibited superior hydration capacity, contributing to delayed gastric emptying of the mixed solid–liquid phase (*t*_1/2_ = 85.1 ± 1.0 min) compared to ADM (*t*_1/2_ = 80.4 ± 1.2 min), indicating increased gastric satiety. Conversely, VFDM showed a faster solid-phase gastric emptying (*t*_1/2_ = 107.2 ± 0.8 min) relative to ADM (*t*_1/2_ = 113.1 ± 2.7 min), likely due to improved texture and rehydration. Both jerky types exhibited progressive particle disintegration; by 180 min, large particles (>2.0 mm) decreased to 16.88% ± 2.63% in ADM and 20.04% ± 0.64% in VFDM (*p* > 0.05). Protein digestibility, measured by SDS-PAGE and the degree of hydrolysis (DH), was significantly higher in VFDM (38.5 ± 3.6%) than in ADM (34.0 ± 0.1%, *p* < 0.05), with VFDM demonstrating more rapid and extensive protein degradation across gastric and intestinal phases. These improvements may be attributed to the porous microstructure and reduced processing-induced protein cross-linking in VFDM, facilitating enhanced enzyme access. Overall, vacuum freeze-drying substantially improved yak jerky protein digestibility, offering the potential for the development of meat-based functional foods targeted at individuals with compromised gastrointestinal function.

## 1. Introduction

The yak (*Bos grunniens*), an iconic bovine species native to the Qinghai–Tibetan Plateau, thrives in the region’s pristine and high-altitude environment. Adapted to harsh alpine conditions, yak meat is naturally rich in high-quality protein (~23.35%), essential amino acids, and unsaturated fatty acids (~45%), while being low in fat (~3.45%) [1]. These nutritional advantages, along with its ecological purity, make yak meat a highly valued and health-conscious food source among consumers [2]. Over the years, various processing techniques have been developed to preserve yak meat, among which high-temperature steaming and natural air-drying are the most commonly employed. However, high-temperature processing is known to denature proteins and promote collagen aggregation, ultimately reducing protein digestibility and limiting nutritional availability [3]. In contrast, traditional air-drying, widely practiced by local communities, leverages the cold, arid climate of late autumn to early winter. This low-temperature method allows water to sublimate directly from frozen meat, better preserving its nutritional content and yielding a distinctive texture and flavor [4].

Air-dried yak meat holds both cultural and practical significance. It is a staple food for Tibetan herders due to its portability, long shelf life, and high nutritional density. However, this method is highly dependent on environmental conditions such as ambient temperature, wind speed, and humidity, which are difficult to control. As a result, the product often suffers from inconsistent texture, color variation, and compromised hygiene due to minimal mechanization and lack of sterile conditions [5]. Similarly, high-temperature drying results in dense texture, poor rehydration capacity, darkened color, and substantial nutrient loss, particularly of thermally sensitive proteins and pigments, all of which negatively impact consumer acceptance and market value [3,6,7].

In meat products, protein quality and digestibility are critical nutritional indicators. However, prolonged or poorly controlled drying processes often accelerate protein oxidation, leading to changes in solubility, gelation behavior, hydrophobicity, and overall amino acid bioavailability [1,8,9]. Studies have shown that during conventional air-drying, protein carbonyl content increases while enzymatic hydrolysis efficiency decreases, indicating a loss of digestible protein over time [10]. Consequently, there is a growing need for advanced drying methods that preserve meat quality while improving digestibility.

In this context, vacuum freeze-drying has emerged as a promising non-thermal preservation technique for improving the digestibility of high-protein foods during *in vitro* digestion [11,12,13,14]. Similar to air-drying, it involves the sublimation of ice, but under vacuum and controlled low-temperature conditions. This process minimizes oxidative damage and preserves the original color, aroma, flavor, and structural integrity of meat, while also reducing drying time. Although the benefits of low temperature or freeze-drying in maintaining the physical and nutritional properties of foods such as pork meat and yellow croaker [15,16] are well-documented, its specific impact on gastrointestinal digestibility, particularly in high-protein foods like yak meat, remains largely unexplored.

Food processing profoundly influences the digestive behavior of food matrices, including the rate and extent of nutrient breakdown. Structural changes in processed meat can alter its interaction with digestive enzymes, affecting key physiological processes such as gastric emptying, the rate at which chyme moves from the stomach into the small intestine, which directly influences nutrient absorption [17]. Although numerous studies have examined the composition, flavor, and traditional processing of yak meat [2,6,8,18], limited attention has been paid to how processing methods affect the dynamic gastrointestinal digestion of yak meat. Most current studies rely on static *in vitro* models, which cannot adequately mimic the dynamic conditions of the human gastrointestinal tract, particularly gastric emptying behavior [3,19].

To address these issues, the present study employs a dynamic *in vitro* human stomach–intestine (DHSI-IV) system to evaluate and compare the gastric emptying profiles, protein hydrolysis efficiency, and particle size distribution of vacuum freeze-dried yak meat (VFDM) and traditional air-dried yak meat (ADM). This is the first systematic investigation using a dynamic digestion model to assess how these two preservation methods influence the bioaccessibility and digestive behavior of yak jerky. The findings will not only provide mechanistic insights into the digestive performance of differently processed yak meat but also offer valuable guidance for developing high-quality, low-temperature dried meat products with improved nutritional functionality.

## 2. Materials and Methods

### 2.1. Materials and Samples Preparation

#### 2.1.1. Materials

Air-dried yak meat (ADM) was sourced from the Tibetan University of Agriculture and Animal Husbandry (TUAH), China. Vacuum freeze-dried yak meat (VFDM) was prepared in the laboratory using fresh yak meat also sourced from TUAH.

#### 2.1.2. Preparation of Yak Jerky

To prepare ADM, fresh yak meat, from which the fascia and fat on the surface were removed, was divided into long strips with a cross-section of approximately 2 cm × 1 cm (width × height) and hung on ropes or wires and exposed to the ambient plateau environment beginning in November, when local temperatures ranged from approximately −4 °C to 14 °C. The meat underwent natural air-drying for approximately 40 days, during which direct sunlight was avoided to prevent quality degradation. Upon completion of the drying process, the jerky was sealed in an airtight container with desiccants and stored at room temperature. The final moisture content was measured as 11.97 ± 0.47% at the time of analysis.

To prepare VFDM, the fascia and fat were first removed from fresh yak meat, and then it was cut into long strips with approximate dimensions of 5 cm × 1 cm × 1 cm (length × width × height). These strips were then frozen at −40 °C for 12 h, followed by freeze-drying in a vacuum freeze-dryer for 7.5 h. The resulting moisture content was 12.23 ± 0.55%. Prior to digestion, the protein content of both samples was determined using the Kjeldahl method [20], with VFDM containing 72.7 g protein/100 g and ADM containing 61 g protein/100 g. After processing, VFDM samples were sealed in airtight containers and stored at room temperature for further use.

### 2.2. Chemicals and Preparation of Simulated Digestive Fluids

#### 2.2.1. Chemicals

Pepsin (P7000) from porcine gastric mucosa, α-amylase (10070) from *Bacillus* sp., pancreatin (P7545) from porcine pancreas, and bile salts (48305) were all purchased from Sigma-Aldrich (St. Louis, MO, USA). All other chemicals and reagents used were of analytical grade and obtained from Sinopharm Chemical Reagent Co., Ltd. (Beijing, China).

#### 2.2.2. Preparation of Simulated Digestive Fluids

Simulated salivary fluid (SSF), simulated gastric fluid (SGF), and simulated intestinal fluid (SIF) were formulated with minor modifications based on previously published protocol [21]. Electrolyte stock solutions for SSF, SGF, and SIF were formulated separately using the following components: KCl (0.5 mol/L), KH_2_PO_4_ (0.5 mol/L), NaHCO_3_ (1 mol/L), NaCl (2 mol/L), MgCl_2_·6H_2_O (0.15 mol/L), and (NH_4_)_2_CO_3_ (0.5 mol/L). Prior to use, the stock solution for SSF was adjusted to pH 7.0, followed by the addition of distilled water, CaCl_2_(H_2_O)_2,_ and α-amylase to achieve a CaCl_2_(H_2_O)_2_ concentration of 1.5 mM and an enzymatic activity of 150 U/mL. The SGF stock solution was adjusted to pH 1.60 and supplemented with water, CaCl_2_(H_2_O)_2,_ and pepsin to reach a CaCl_2_(H_2_O)_2_ concentration of 0.15 mM and an enzymatic activity of 4000 U/mL. Similarly, the SIF stock solution was adjusted to pH 6.8, and water, CaCl_2_(H_2_O)_2_, pancreatin, and bile salts were added to achieve a CaCl_2_(H_2_O)_2_ concentration of 0.6 mM, a final pancreatin activity of 200 U/mL and a bile salt concentration of 20 mM.

### 2.3. Measurement of Water Absorption Capacity of Yak Jerky

Individually, 1 g samples of ADM and VFDM were weighed. These samples were meticulously cut into pieces of comparable dimensions and then immersed in 20 mL of a gastric fluid-simulating liquid electrolyte solution devoid of pepsin, ensuring that the solution could entirely submerge the samples. For each sample type, five replicates were prepared. These replicates were, respectively, immersed for durations of 30, 60, 120, 150, and 180 min. Subsequently, they were retrieved, and the surface moisture was promptly blotted off before being weighed. The moisture absorption ratio (%) was calculated as the ratio of mass gain after soaking to the initial dry mass of the sample.

### 2.4. In Vitro Dynamic Digestion Using the DHSI-IV System

**Figure 1:**
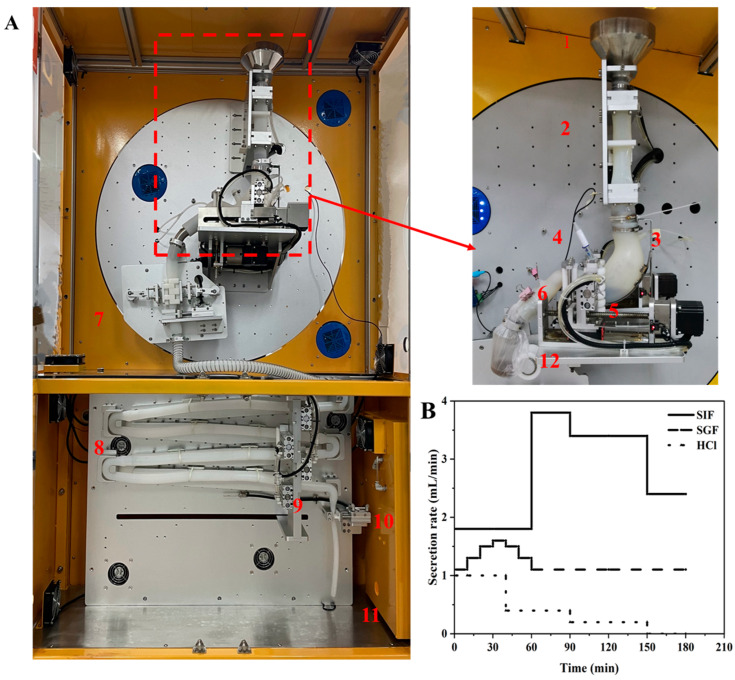
Photograph of the DHSI-IV system (**A**) and flow rates of simulated gastric fluid (SGF), simulated intestinal fluid (SIF), and gastric acid (HCl) during digestion (**B**). (1) Sample funnel; (2) esophageal model; (3) stomach model; (4) pH probe; (5) peristalsis contraction rollers; (6) pylorus; (7) duodenal peristalsis device; (8) small intestine model; (9) alternate peristaltic roller; (10) intestinal valve; (11) intestinal chyme outlet; (12) gastric chyme outlet.

The gastrointestinal digestion characteristics of VFDM and ADM were investigated using a dynamic *in vitro* human stomach–intestine (DHSI-IV) system, developed by Xiao Dong Pro-health Instrumentation Co., Ltd. (Suzhou, China), as illustrated in Figure 1. As previously described [22], the DHSI-IV system consists of a J-shaped soft silicone stomach model, an approximately 4 m long silicone small intestine model, a mechanical peristalsis simulation unit, a simulated digestive fluid delivery system, a control interface, and a heating and temperature-maintaining module. This system has been widely employed in simulating the *in vitro* digestion of various food and pharmaceutical matrices [23,24].

For each experimental trial, 50 g of ground yak jerky was used, divided into five 10 g portions. Each portion was pre-mixed with 10 mL of simulated saliva at 37 °C for 1 min to mimic oral processing. Then, 20 mL of water was added, and the mixture was introduced into the injection funnel. Each portion took approximately 2 min to pass through the esophageal model into the stomach chamber. The five portions were injected sequentially at 2 min intervals, resulting in a total input of 50 g jerky, 50 mL simulated saliva, and 100 mL water, corresponding to an injection rate of approximately 20 g/min. Simulated digestion began immediately upon the first injection. At the onset, 30 mL of digestive fluid was automatically introduced into the stomach model to mimic the fasting state of the human stomach [25]. During the digestion process, the secretion of gastric and intestinal fluid simulants was precisely regulated by a syringe pump to ensure continuous secretion. The secretion of gastric and intestinal fluids was controlled precisely using a syringe pump, maintaining flow rates of 1.1–1.6 mL/min and 1.8–2.4 mL/min, respectively, as illustrated in Figure 1. These rates were adjusted based on human physiological data for gastric secretion [26] and gastric emptying [27]. To replicate *in vivo* gastric acidification dynamics [28], simulated gastric acid (1 M, HCl) was secreted into the stomach model at a rate of 0.2–1 mL/min, resulting in a gradual intragastric pH decrease from approximately 5.6 to 1.2 during the 180 min digestion period. A built-in microprobe pH sensor continuously monitored intragastric pH throughout the experiment. The mechanical motion system applied vertical and horizontal compression forces to the gastric wall via rollers positioned on either side of the stomach model, mimicking the longitudinal and transverse contractions of human gastric peristalsis at a frequency of three times per minute. The entire gastrointestinal digestion process, including both gastric and intestinal phases, was simulated using the DHSI-IV system. Samples were collected at 60, 120, and 180 min for subsequent analysis of digestion products.

### 2.5. Measurement of Particle Size Distribution of Gastric Digesta

The particle size distribution of gastric digesta from yak jerky prepared by air drying (ADM) and freeze-drying (VFDM) during digestion in the DHSI-IV system was evaluated using the wet sieving method. This technique is widely used for determining the coarse particle size distribution of products resulting from simulated oral and gastric digestion due to its efficiency and simplicity [29]. In this study, we analyzed the particle size distributions of yak jerky pellets after simulated oral digestion, as well as the chyme at 1 h, 2 h, and 3 h intervals following gastric digestion. At each time point, the gastric contents were thoroughly mixed. A 50–100 g sample was then taken and gently agitated with 1000 mL of water to ensure uniform dispersion. The particles were sequentially sieved through 2 mm and 1 mm meshes, and this process was repeated twice. The solids retained on the sieves were collected and dried in an oven at 105 °C for 24 h. The solid content of the fraction passing through the 1 mm sieve was measured, and the total mass of the gastric digesta passing through the sieves was carefully recorded. These data were used to calculate the total dry weight of particles smaller than 1 mm. The particle distribution of the chyme remaining in the gastric model was analyzed using the same procedure.

### 2.6. Determination of Gastric Emptying Rate

The gastric emptying rates of VFDM and ADM during simulated gastric digestion were assessed using the DHSI-IV system, as previously described [25]. During the digestion process, gastric discharges (chyme) were collected at hourly intervals and weighed. Subsequently, each sample was dried at 105 °C for 48 h to determine both the total and solid (dry matter) gastric emptying rates. The total (including both solid and liquid fractions) gastric emptying rate was calculated by expressing the cumulative weight of the discharged chyme at each time point as a percentage of the total input into the system, which included yak jerky, water, SGF, and hydrochloric acid (HCl). The solid gastric emptying rate was determined by dividing the mass of dry matter in the discharged chyme by the dry mass of the initially fed yak jerky (approximately 44 g). The total and solid gastric retention ratios at each time point were calculated using the following equations [30]:

(1) $$\mathrm{Total}\;\mathrm{gastric}\;\mathrm{retention}\;\mathrm{ratio}\;(\%)=\frac{m_{t}}{m_{o}+m_{SGF,t}+m_{HCl,t}}\times 100\%$$

where $m_{t}$ is the mass (g) of the digesta remaining in the stomach model at time *t* (min), $m_{o}$ is the mass (g) of the initial total sample loaded into the stomach model (including SSF, yak jerky, and water), and $m_{SGF,t}$ and $m_{HCl,t}$ are the masses of SGF and HCl delivered into the stomach model up to time *t* (min), respectively.

(2) $$\mathrm{Solid}\;\mathrm{gastric}\;\mathrm{retention}\;\mathrm{ratio}\;(\%)=\frac{m_{dry,t}}{m_{dry,0}}\times 100\%$$

where $m_{dry,t}$ is the dry mass (g) of the digesta remaining in the stomach model at time *t* (min) and $m_{dry,0}$ is the dry mass (g) of the initially fed yak jerky (44 g).

The gastric emptying of chyme was analyzed using the modified Elashoff’s power-exponential model, which is widely used for quantifying key gastric emptying parameters, such as the lag phase time (*t*_la_*_g_*) and the half-time (*t*_1/2_). This model allows for a robust quantitative comparison of gastric emptying dynamics across different conditions. The model equations are as follows [31]:

(3) *y*(t) = 1 − (1 − e^−kt^)*^β^*

(4) $$t_{1/2}=(-\frac{1}{k})\times \ln(1-0.5^{\frac{1}{\beta }})$$

(5) $$t_{\mathrm{tag}}=\frac{ln\beta }{k}$$

where *y*(t) represents the gastric retention ratio at time *t* (min), *k* is the emptying rate constant (1/min), and *β* is a parameter determined by the curvature of the emptying curve. All experiments were conducted in triplicate to ensure statistical reliability and reproducibility.

### 2.7. Analysis of Protein Hydrolysis During Simulated Dynamic Digestion

The changes in serine-NH_2_ concentration during simulated dynamic digestion in the DHSI-IV system were analyzed using the o-phthaldialdehyde (OPA) method, with slight modifications from a previously reported protocol [32]. Briefly, an equal volume of the product obtained from the dynamic digestion was mixed with a 10% trichloroacetic acid (TCA) solution to inactivate digestive enzymes and precipitate undigested large protein aggregates. The mixture was allowed to stand for 20 min, after which the supernatant was collected by centrifugation at 1000 rpm for 10 min at 4 °C. Serine (Merck Art. 7769) was used as the standard. The supernatant (200 μL) was then reacted with a freshly prepared OPA solution (1.5 mL), and the variation in serine-NH_2_ content was quantified at 340 nm using a spectrophotometer (Mapada Co., Ltd., Shanghai, China). To determine the maximum theoretical release of free amino groups, a 5 g sample was treated with 110 mL of simulated digestive fluid and hydrolyzed for 24 h. The total free amino content after complete hydrolysis was measured using the same procedure. The degree of hydrolysis (DH), representing the percentage of cleaved peptide bonds, was calculated using the following equation:

(6) $$\mathrm{DH}\;(\%)=\frac{\left[-NH_{2\left(t\right)}]-[-{NH}_{2(t-0)}\right]}{\left[-{NH}_{2\left(\infty \right)}\right]}\times 100\%$$

where [−NH_2(t)_] is the amino group concentration after digestion at time *t*, [−NH_2(t − 0)_] is the initial (*t* = 0) amino group content in the digestion fluid (baseline), and [−NH_2(__∞__)_] is the total amino group content after complete hydrolysis (24 h).

### 2.8. SDS-PAGE Analysis of Digestion Products

Proteolysis of yak jerky prepared using both drying methods during simulated gastrointestinal digestion was assessed via SDS-PAGE analysis according to an established protocol [33]. At 60, 120, and 180 min of the simulated digestion process, 2 mL samples each of gastric and intestinal digestion products were collected. These samples were then centrifuged at 10,000 rpm for 10 min at 4 °C, and the enzymes were inactivated by boiling the samples at 100 °C for 3 min. The gastric digestion products were diluted fivefold before analysis, while the intestinal digestion products were analyzed without dilution. After the sample treatment, MES SDS electrophoresis buffer was added to each sample, and electrophoresis was conducted on 4–12% Bis-Tris gels using a buffer at 200 V for 54 min. Upon completion, the gel was stained with Thomas Blue R-250 (Bio-Rad, Hercules, CA, USA) solution for 4 h. The gel was then destained using a solution composed of 7.5% acetic acid, 10% ethanol, and 82.5% distilled water (*v*/*v*/*v*, mixed at a 1:5:4 ratio) for 3.5 h. Finally, the gel was scanned using a Bio-Rad Universal Hood II ChemiDoc Molecular Imager XRS+ (Bio-Rad, Hercules, CA, USA).

### 2.9. Statistical Analysis

All data are presented as the mean ± standard deviation from at least two independent experiments. The normality of data distribution and homogeneity of variances were verified using the Shapiro–Wilk and Levene’s tests, respectively. For comparisons involving only two groups (VFDM and ADM), Student’s *t*-test was applied. In cases where more than two groups were compared, one-way analysis of variance (ANOVA) followed by Tukey’s post hoc test was used. Statistical significance was considered at *p* < 0.05. All analyses were performed using SPSS version 22.0 (IBM, Armonk, NY, USA).

## 3. Results and Discussion

### 3.1. Water Absorption During Static Soaking

**Figure 2:**
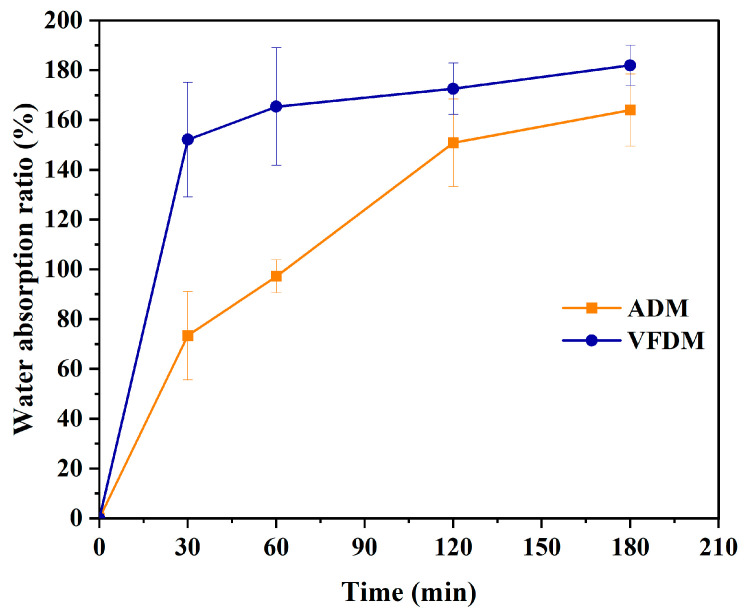
Changes in water absorption ratio of air-dried (ADM) and vacuum freeze-dried (VFDM) yak jerky during static soaking in SGF for 180 min (*n* = 3).

To compare the water absorption capacities of the two types of yak jerky, samples were soaked in SGF without pepsin. As shown in Figure 2, both types of jerky absorbed water over time, but with markedly different kinetics and extents of hydration. The VFDM samples exhibited a rapid increase in water uptake, reaching approximately 152.09% of their initial weight within the first 30 min. This sharp increase was followed by a plateau, indicating that the porous matrix formed during freeze-drying rapidly absorbed water to near saturation. The freeze-drying process preserves the open muscle fiber structure and internal porosity, enhancing capillary-driven fluid infiltration and enabling faster and more extensive rehydration [34,35]. In contrast, ADM samples absorbed water more slowly and progressively, with hydration continuing throughout the entire 180 min soaking period, with statistically different in the first 60 min (*p* < 0.05, Table S1 in Supplementary Material). At the end of soaking, VFDM and ADM had absorbed approximately 181.92% and 163.99% of their initial weights, respectively. The slower hydration and reduced expansion in ADM are likely due to structural changes induced by extended air-drying, which causes muscle fiber shrinkage, tightens inter-fiber gaps, and promotes protein cross-linking, creating a denser and less permeable tissue matrix [10]. The markedly different water absorption behaviors reflect the influence of the drying method on the jerky’s microstructure. The expanded, sponge-like texture of VFDM increases the available surface area and facilitates deeper fluid penetration, which may subsequently enhance enzyme accessibility and digestion efficiency. In contrast, the compact, rigid network in ADM restricts both hydration and enzymatic diffusion, potentially limiting proteolytic breakdown during gastrointestinal digestion [15].

### 3.2. Apparent Morphology During Dynamic Digestion

**Figure 3:**
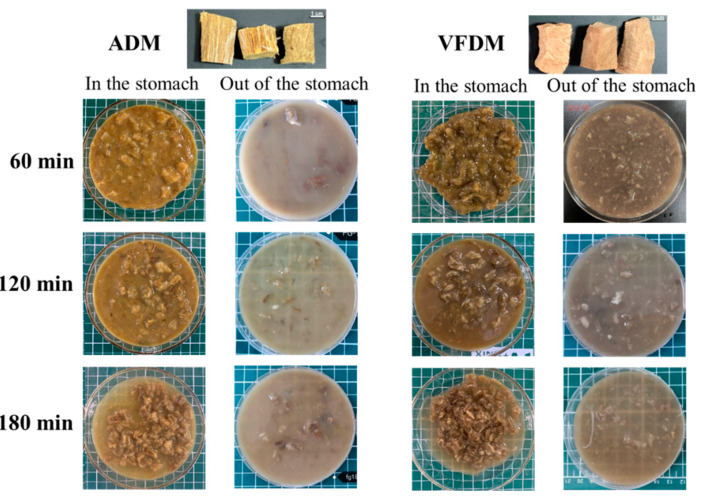
Apparent morphology of gastric digesta (including both chyme retained in the stomach and that emptied from the stomach) of air-dried (ADM) and vacuum freeze-dried (VFDM) yak jerky collected at different time points (60, 120, and 180 min) during simulated gastric digestion (*n* = 3).

As shown in Figure 3, distinct differences in coloration were observed between ADM and VFDM prior to digestion. ADM samples exhibited a dark yellow to brownish color, consistent with the browning reactions and pigment oxidation that occur during prolonged air-drying. In contrast, VFDM samples retained a lighter red hue, indicative of minimal myoglobin degradation. This color preservation is attributed to the low-temperature, low-pressure environment of freeze-drying, which reduces oxidative reactions and limits the Maillard browning that typically accompanies heat-based dehydration. These observations are consistent with previous findings that longer air-drying times correlate with reduced lightness and brightness of dried meat products [10,15].

To visualize the *in vitro* digestive progression of both jerky types, morphological changes in gastric digesta (including both chyme retained in the stomach and that emptied from the stomach) were documented at sequential time points during the dynamic digestion process (Figure 3). After 60 min of digestion, clear differences in chyme structure emerged. The VFDM samples demonstrated greater fluid-binding capacity, forming larger, cohesive intragastric boluses. The gastric contents were characterized by a heterogeneous mixture of solids and bound fluids, suggesting efficient hydration and structural softening of the matrix. This is likely facilitated by the porous and sponge-like texture imparted by freeze-drying, which enhanced the infiltration of gastric fluid and promoted chyme aggregation [35]. In contrast, ADM samples showed less cohesive chyme morphology, with a visibly higher proportion of unbound liquid and a more paste-like expelled texture. This behavior reflects the dense, compact tissue network formed during air-drying, which resisted fluid penetration and reduced water retention (Figure 2). The limited structural loosening observed in ADM may impede the formation of uniform chyme aggregates, potentially affecting the subsequent enzymatic interactions and transit.

At 120 min, the morphological differences between the two chyme types began to converge, with both samples showing more uniform textures and moisture distribution. This convergence aligns with the progressive hydration observed during the water absorption phase (Section 3.1), suggesting that extended exposure to digestive fluids partially mitigated the initial structural disparities caused by different drying methods. Chyme samples collected from the terminal outlet of the stomach model at 60 min revealed no visible large food particles for either jerky type. This observation likely reflects the gastric retention effect common in solid food digestion [36], wherein larger undigested particles were delayed in gastric emptying. By 120 min and beyond, small residual fragments and semi-digested particles were gradually released, indicating progressive breakdown and chyme flow through the gastrointestinal model.

### 3.3. Gastric pH

**Figure 4:**
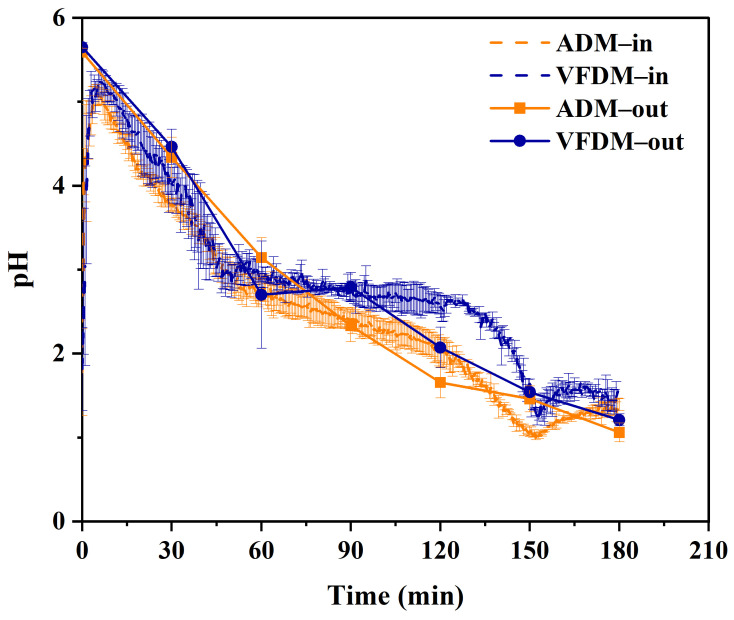
pH profiles of gastric digesta (including chyme retained in the stomach and emptied from the stomach) for air-dried (ADM) and vacuum freeze-dried (VFDM) yak jerky during dynamic gastric digestion (*n* = 3). Intragastric pH was continuously monitored using an integrated micro pH probe, while the pH of the emptied digesta was measured with a standard pH meter.

The dynamic changes in intragastric pH during simulated gastric digestion of VFDM and ADM yak jerky are presented in Figure 4. Following simulated oral digestion, the initial pH values of the food boluses were 5.65 ± 0.06 for VFDM and 5.59 ± 0.05 for ADM. Upon entry into the stomach model, a sharp rise in pH was observed, reflecting the buffering action of the food matrix [37]. As simulated gastric fluid and acid were secreted and gastric emptying proceeded, the pH of both samples rapidly declined to approximately 3.3 within the first 47 min, followed by a slower decrease to ~1.6, which corresponds to the fasting-state pH of the simulated gastric fluid. This acidification reflects the onset of active digestion and the optimal conditions for pepsin activation.

Throughout the digestion process, VFDM consistently exhibited a slightly higher intragastric pH compared to ADM, although these differences were not statistically significant at most time points (see Table S2 in Supplementary Material). This phenomenon can be primarily attributed to the superior water absorption capacity of the VFDM matrix (Figure 2), which results from its relatively porous and soft texture. The VFDM samples more readily absorbed gastric juices and acid, allowing deeper fluid penetration and promoting the release of more nutrients, particularly proteins and peptides, into the digesta [25,38]. These released compounds contributed to a higher buffering capacity in VFDM, which in turn necessitated greater acid secretion to achieve pH levels comparable to ADM [39]. In contrast, ADM samples exhibited more distinct solid–liquid separation, with less fluid retained in the matrix and a greater proportion of free gastric fluid contributing to the pH measurement. Consequently, the pH probe, which captures the average pH of the solid–liquid mixture, recorded a lower pH for ADM due to its relatively limited buffering action. These structural and physicochemical differences between the jerky types not only influenced pH dynamics but may also have implications for the rate and efficiency of gastric digestion. These findings are consistent with previous reports on intragastric pH trends during the digestion of meat products [40] and highlight how food texture and water interaction properties can modulate digestive conditions and nutrient bioaccessibility.

### 3.4. Particle Size Distribution

One of the primary physiological functions of the stomach is to mechanically break down ingested food into smaller particles of chyme, thereby facilitating its controlled passage into the duodenum to initiate intestinal digestion [41]. Particle size plays a pivotal role in this process, as smaller particles typically exhibit enhanced surface area, leading to more efficient enzymatic digestion and accelerated gastric emptying rates [42]. Furthermore, the physical disintegration of food in the stomach directly influences nutrient bioaccessibility and the overall digestive kinetics. In this study, we evaluated the particle size distribution of ADM and VFDM yak jerky samples throughout dynamic gastric digestion using the DHSI-IV system. Particle size was categorized into three groups: small (0–1.0 mm), medium (1.0–2.0 mm), and large (2.0–10.0 mm) [43], and measurements were taken at 0, 60, 120, and 180 min (see Table S3 in the Supplementary Material).

**Figure 5:**
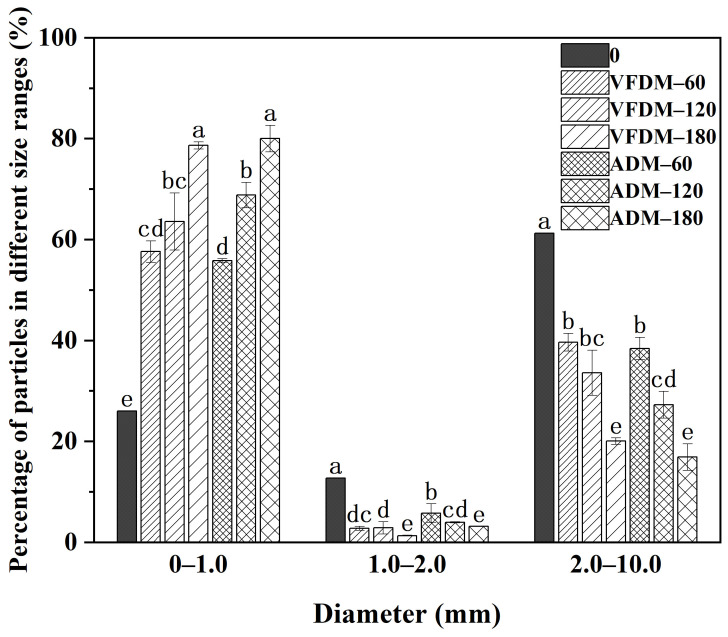
Particle size distribution profile of the air-dried (ADM) and vacuum freeze-dried (VFDM) yak jerky during dynamic digestion in the DHSI-IV system (*n* = 3). Data within the same particle diameter range marked with different letters indicate significant differences at *p* < 0.05. Error bars represent standard deviations.

As shown in Figure 5, at the onset of gastric digestion (0 min), a high proportion (60.79 ± 1.96%) of large particles (>2.0 mm) was observed in both ADM and VFDM samples, indicating their relatively coarse texture post-oral digestion. As digestion progressed, significant particle breakdown was evident in both samples. The proportion of large particles steadily declined, while that of small particles (<1.0 mm) increased, reflecting the mechanical grinding and enzymatic actions of the gastric environment. At 180 min, large particle content dropped substantially to 16.88 ± 2.63% in ADM and 20.04 ± 0.64% in VFDM. These findings underscore the effective breakdown of both jerky types under simulated gastric conditions, with no statistically significant difference in overall disintegration patterns between ADM and VFDM across the tested time points (*p* > 0.05). It is worth noting that although VFDM displayed slightly larger residual particles at later stages, this did not translate into significant differences in particle size distribution.

### 3.5. Gastric Emptying Rate

**Figure 6:**
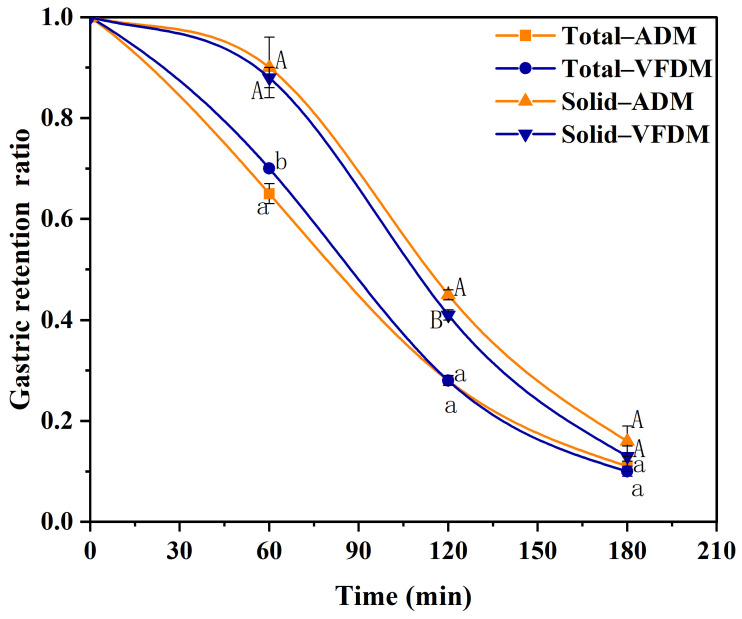
Gastric emptying profiles of total (mixed solid–liquid materials) and solid (dry basis) fractions of air-dried (ADM) and vacuum freeze-dried (VFDM) yak jerky during dynamic digestion in the DHSI-IV system (*n* = 3). Data were fitted using the modified Elashoff’s power exponential model. The data marked in the same digestion time points with different uppercase (or lowercase) letters represent a significant difference at *p* < 0.05.

The gastric emptying rate of the ADM and VFDM yak jerky samples was evaluated using the DHSI-IV dynamic digestion system, with the results presented as intragastric retention curves in Figure 6. The power-exponential model proposed by Elashoff was employed to fit the gastric emptying data, providing key parameters such as the half-emptying time (*t*_1/2_), lag time (*t*_lag_), and the rate constants *k* and *β*, which are summarized in Table 1. The gastric emptying kinetics were all well described by the Elashoff model, with coefficients of determination (*R^2^*) ranging from 0.995 to 1.000 across all groups, indicating excellent model fit.

For the total gastric emptying (including both solid and liquid fractions), ADM exhibited a slightly faster overall emptying profile during the early digestion stage. The total *t*_1/2_ for ADM was 80.4 ± 1.2 min, significantly lower (*p* < 0.05) than that of VFDM (85.1 ± 1.0 min). Similarly, the total *t*_lag_ for ADM was 50.4 ± 5.5 min, compared to 60.9 ± 0.03 min for VFDM. Despite VFDM having a slightly higher rate constant *k* than ADM, the higher *β* value of VFDM (3.26 ± 0.12) versus ADM (2.40 ± 0.43) suggests a more delayed onset but sharper acceleration in emptying after the lag phase [22,39]. These findings imply that the faster early-phase emptying in ADM is primarily attributed to the rapid discharge of unbound liquid, a consequence of ADM’s weak moisture absorption capacity (Figure 2). The digestive fluids were less retained in the ADM matrix, leading to accelerated early-phase emptying of the liquid fraction, including dissolved nutrients and small chyme particles. In contrast, VFDM, with its superior fluid-binding and water absorption capability, retained more digestive fluid during the initial phase. This interaction delayed liquid emptying, explaining the longer *t*_lag_ and *t*_1/2_ values observed. As digestion progressed and pepsin activity softened the matrix, VFDM gradually released more of the solid–liquid chyme mixture, equalizing the overall gastric emptying behavior between the two samples by 120 min.

When solid-phase gastric emptying was analyzed independently, the pattern reversed. VFDM displayed a faster solid emptying rate than ADM, as evidenced by a significantly lower half-emptying time (*t*_1/2_ = 107.2 ± 0.8 min) compared to ADM (*t*_1/2_ = 113.1 ± 2.7 min, *p* < 0.05). The lag time for solid emptying was slightly lower in VFDM than in ADM, although this difference was not statistically significant. Both samples demonstrated a long lag phase, consistent with typical gastric retention patterns where solids were retained longer while the liquid was exponentially emptied [31]. This enhanced solid-phase emptying in VFDM can be attributed to its softer texture and improved hydration, which promote better breakdown and mixing, resulting in smaller, more flowable particles that can exit the gastric model more efficiently [22,30,43]. ADM, with its firmer and less hydrated matrix, resisted breakdown, thereby prolonging its solid-phase gastric retention time.

In terms of nutritional implications, the longer gastric retention of VFDM (as reflected in its higher total *t*_lag_ and *t*_1/2_) suggests it may contribute to greater and more sustained gastric distension, thereby enhancing satiety signals [44]. This could offer advantages in appetite regulation or weight management applications. Meanwhile, its more efficient solid-phase emptying supports improved digestibility once initial breakdown is achieved.

### 3.6. Degree of Hydrolysis During Dynamic Gastrointestinal Digestion

**Figure 7:**
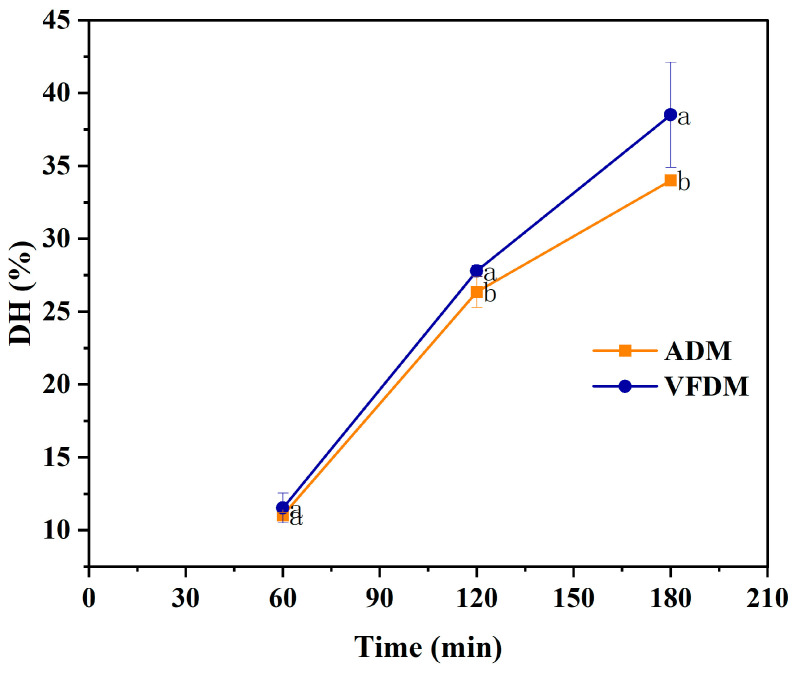
Degree of protein hydrolysis (DH, %) of the air-dried (ADM) and vacuum freeze-dried (VFDM) yak jerky during dynamic digestion in the DHSI-IV system (*n* = 3). Different lowercase letters at the same time point indicate statistically significant differences between ADM and VFDM (*p* < 0.05).

The dynamic digestive behavior of yak jerky was further characterized by continuously monitoring the degree of protein hydrolysis (DH, %) using the DHSI-IV system, as illustrated in Figure 7. In this system, gastric and intestinal digestion proceed simultaneously, providing a more physiologically relevant simulation of the human digestive tract. The DH value reflects the extent of peptide bond cleavage and thus serves as a direct indicator of protein digestibility and bioaccessibility. Over the 180 min digestion period, the VFDM consistently exhibited a higher DH compared to the ADM. At the end of digestion, VFDM reached a peak DH of approximately 38.5 ± 3.6%, while ADM only attained 34.0 ± 0.1% (*p* < 0.05), indicating that VFDM was more efficiently hydrolyzed during digestion.

The superior hydrolysis performance of VFDM is likely attributed to its preserved porous microstructure and improved rehydration capacity, which enhance enzymatic accessibility. In contrast, thermal processing during air-drying can lead to protein cross-linking and side-chain modifications, limiting enzyme-substrate interaction and thereby reducing hydrolytic efficiency. This observation is consistent with earlier studies showing that drying methods can induce structural changes in proteins. In particular, they may lead to the formation of intra- and intermolecular disulfide bonds. These structural alterations can either expose or conceal proteolytic cleavage sites, depending on the extent of protein oxidation [9,45,46]. Moderate oxidation, typically triggered by processing stress or exposure to reactive oxygen species, may unfold protein tertiary structures and expose buried peptide bonds, thus facilitating enzymatic cleavage. However, excessive oxidation tends to result in irreversible protein aggregation or cross-linking, which hinders protease binding and reduces digestibility. The relatively low DH observed in ADM supports the notion that such cross-linking dominated in the air-dried sample, diminishing its susceptibility to enzymatic breakdown.

### 3.7. SDS-PAGE

**Figure 8:**
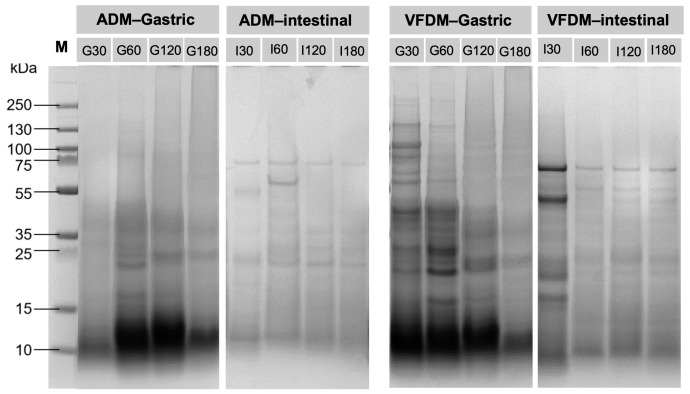
SDS-PAGE patterns of yak jerky digesta from two drying methods—air-drying (ADM) and vacuum freeze-drying (VFDM)—at different time points during dynamic *in vitro* gastric and intestinal digestion in the DHSI-IV system (*n* = 2). Protein degradation was monitored under reducing conditions. Lane M: protein marker; lanes G30–G180: gastric digesta at 30, 60, 120, and 180 min; lanes I30–I180: intestinal digesta at 30, 60, 120, and 180 min. Band intensities and molecular weights indicate the extent of enzymatic hydrolysis at each phase.

SDS-PAGE was employed to analyze the degradation profiles of yak jerky proteins during simulated dynamic gastric and intestinal digestion (Figure 8). The electrophoretic patterns revealed that different drying methods influenced the protein digestion efficiency throughout the gastrointestinal phases. In ADM samples, digestion progressed relatively slowly in the gastric phase. At 30 min, only faint bands were visible, primarily above 15 kDa, suggesting that proteins remained partially intact, possibly due to the dense matrix formed during air-drying [3,10,47]. As digestion advanced, bands below 15 kDa began to appear at 60 min and became more prominent by 120 and 180 min, indicating gradual pepsin-mediated hydrolysis [30,39]. However, the breakdown remained less extensive compared to VFDM, reflecting limited enzyme accessibility to substrate proteins in the compact ADM matrix. During the intestinal phase of ADM digestion, further hydrolysis occurred with most bands shifting below 15 kDa, particularly from 60 min onward. At 180 min, only low molecular weight peptides (<10 kDa) were visible, indicating effective digestion by pancreatic enzymes. The delayed appearance and gradual accumulation of low-molecular-weight bands support the notion that gastric digestion in ADM was slower, resulting in a reduced substrate load entering the intestinal phase initially.

In contrast, VFDM samples exhibited markedly enhanced digestion kinetics. By 30 min of gastric digestion, broad and intense protein bands ranging from 10 to 70 kDa were evident, suggesting rapid and extensive proteolysis. This can be attributed to the porous and rehydratable structure of the freeze-dried jerky, which promotes better penetration of digestive enzymes [34,38]. Protein fragments continued to accumulate and diversify during the 60–180 min gastric digestion period, with a significant portion of peptides falling below 15 kDa by 180 min. Subsequent intestinal digestion of VFDM samples showed a progressive reduction in band intensity and molecular weight. At 60 min, peptides below 15 kDa dominated the gel, and by 180 min, most bands fell below 10 kDa, indicating almost complete enzymatic breakdown. Compared to ADM, VFDM samples exhibited a smoother transition between gastric and intestinal phases, reflecting more efficient gastric emptying and hydrolysis dynamics in the *in vitro* system. These protein degradation dynamics of ADM and VFDM samples are consistent with previous findings on yak meat prepared using various cooking methods and subjected to *in vitro* static digestion [3].

In summary, protein digestibility, as assessed by SDS-PAGE and the degree of hydrolysis (DH), consistently favored VFDM. At 180 min, VFDM achieved a significantly higher DH than ADM, reflecting more extensive enzymatic breakdown. This was corroborated by SDS-PAGE analysis, which showed a more rapid and complete degradation of VFDM proteins across both gastric and intestinal phases. The superior digestibility of VFDM is likely attributed to its more open and porous matrix structure, enhanced hydration properties, and reduced processing-induced cross-linking. These findings are consistent with particle size (Figure 5) and moisture retention (Figure 2) data, which further support improved digestive accessibility in the freeze-dried matrix.

## 4. Conclusions

This study employed the DHSI-IV system to systematically compare the digestive behavior of yak jerky prepared via air-drying (ADM) and vacuum freeze-drying (VFDM). The results demonstrated that the drying method significantly affected key physicochemical and digestive parameters, which in turn influenced overall protein digestibility. VFDM exhibited superior hydration properties and water-binding capacity, which promoted more uniform softening and enhanced enzyme penetration during digestion. These hydration differences directly influenced early-phase gastric emptying: ADM displayed a faster initial emptying rate of the liquid fraction due to poor moisture absorption, while VFDM retained more fluid, delaying emptying but likely contributing to increased gastric satiety. In contrast, VFDM achieved faster solid-phase gastric emptying, likely due to its softer, more rehydratable texture facilitating chyme flow. Both jerky types underwent progressive particle disintegration under simulated gastric conditions, with large particles (>2.0 mm) significantly decreased after 180 min. Although VFDM retained slightly larger particles, the differences were not statistically significant, indicating a comparable mechanical breakdown in the stomach across treatments. Protein digestibility, as assessed by SDS-PAGE and the degree of hydrolysis, consistently favored VFDM. At 180 min, VFDM achieved a significantly higher DH than ADM, reflecting more extensive enzymatic breakdown. This was corroborated by gel electrophoresis, where VFDM proteins degraded more rapidly and extensively throughout both gastric and intestinal phases. These enhancements in VFDM digestion likely stem from its porous microstructure, better rehydration, and reduced processing-induced protein cross-linking, factors that collectively improve enzyme accessibility and substrate conversion. In conclusion, vacuum freeze-drying offers clear digestive advantages over conventional air-drying in yak jerky, enhancing moisture absorption, modulating gastric emptying dynamics, and promoting protein hydrolysis. These insights may guide the development of meat-based functional foods tailored for populations with altered digestive capacity, such as the elderly or those with compromised gastrointestinal function.

## Figures and Tables

**Table 1:** Gastric emptying parameters based on the modified Elashoff’s power exponential model ^1,2^.

Sample	*k* (1/min)	*β*	*t*_1/2_ (min)	*t*_lag_ (min)	*R* ^2^
Total-ADM	0.0171 ± 0.0017 ^a^	2.4 ± 0.43 ^b^	80.41 ± 1.15 ^d^	50.37 ± 5.54 ^c^	0.999
Total-VFDM	0.0194 ± 0.0006 ^a^	3.26 ± 0.12 ^b^	85.12 ± 0.98 ^c^	60.87 ± 0.03 ^b^	0.999
Solid-ADM	0.0207 ± 0.0048 ^a^	7.52 ± 4.45 ^a^	113.1 ± 2.7 ^a^	91.94 ± 9.18 ^a^	0.995
Solid-VFDM	0.0214 ± 0.0019 ^a^	6.61 ± 1.28 ^a^	107.2 ± 0.83 ^b^	87.54 ± 1.37 ^a^	1

^1^ The data are expressed by mean ± standard deviations based on triplicate measurements for each sample. ^2^ The data marked in the same column with different lowercase letters represent a significant difference at *p* < 0.05.

## Data Availability

The original contributions presented in the study are included in the article/Supplementary Material, further inquiries can be directed to the corresponding authors.

## Supplementary Materials

The following supporting information can be downloaded at: https://www.mdpi.com/article/10.3390/foods14122086/s1. Table S1.Water absorption ratio of the two-type yak jerky in gastric juice (without pepsin). Table S2. pH of the gastric emptied digesta. Table S3. Percentage of particles in different size ranges of the air-dried (ADM) and vacuum freeze-dried (VFDM) yak jerky during dynamic digestion in the DHSI-IV system.
