# Supplementary material for: Comparative Gastrointestinal Digestion Dynamics of Air-Dried and Freeze-Dried Yak Jerky: Insights from a Dynamic In Vitro Human Stomach–Intestine (DHSI-IV) System
Source: Foods. 2025 Jun 13;14(12):2086. doi: 10.3390/foods14122086 (PMC12192123; doi:10.3390/foods14122086)
Supplement: Supplementary file 1 [file foods-14-02086-s001.zip › foods-3668321-supplementary.pdf]

Supplementary Material

Table S1. Water absorption ratio of the two-type yak jerky in gastric juice (without pepsin).

Sample	30 min	60 min	120 min	180 min
ADM	73.36 ± 17.71^{a}	97.19 ± 6.64^{a}	150.79 ± 17.64^{a}	163.99 ± 14.51^{a}
VFDM	152.09 ± 23.01^{b}	165.38 ± 23.63^{b}	172.54 ± 10.29^{a}	181.92 ± 8.08^{a}

[1] The data are expressed by mean ± standard deviations based on triplicate measurements for each sample.

[2] The data marked in the same column with different lowercase letters represent significantly difference at $p < 0.05$ (n=3).

Table S2. pH of the gastric emptied digesta.

Sample	0 min	30 min	60 min	90 min	120 min	150 min	180 min
VFMD	5.65 ± 0.06^{a}	4.46 ± 0.21^{a}	2.7 ± 0.64^{a}	2.79 ± 0.17^{a}	2.07 ± 0.24^{a}	1.54 ± 0.03^{b}	1.21 ± 0.07^{b}
ADM	5.59 ± 0.05^{a}	4.34 ± 0.24^{a}	3.14 ± 0.24^{a}	2.33 ± 0.19^{b}	1.66 ± 0.18^{a}	1.46 ± 0.16^{b}	1.06 ± 0.11^{b}

[1] The data are expressed by mean ± standard deviations based on triplicate measurements for each sample.

[2] The data marked in the same column with different lowercase letters represent significantly difference at $p < 0.05$ (n=3).

Table S3. Percentage of particles in different size ranges of the air-dried (ADM) and vacuum freeze-dried (VFDM) yak jerky during dynamic digestion in the DHSI-IV system.

Sample	0-1.0 mm	1.0-2.0 mm	2.0-10.0 mm
0	18.82 ± 1.83^{e}	21.22 ± 2.35^{a}	60.79 ± 1.96^{a}
VFDM-60	57.6 ± 2.13^{cd}	2.76 ± 0.38^{dc}	39.64 ± 1.75^{b}
VFDM-120	63.56 ± 5.68^{bc}	2.85 ± 1.2^{d}	33.59 ± 4.47^{bc}
VFDM-180	78.65 ± 0.7^{a}	1.31 ± 0.07^{e}	20.04 ± 0.64^{e}
ADM-60	55.82 ± 0.36^{d}	5.78 ± 1.83^{b}	38.4 ± 2.2^{b}
ADM-120	68.81 ± 2.52^{b}	3.94 ± 0.12^{cd}	27.25 ± 2.64^{cd}
ADM-180	80 ± 2.63^{a}	3.13 ± 0^{e}	16.88 ± 2.63^{e}

[1] The data are expressed by mean ± standard deviations based on triplicate measurements for each sample.

[2] Data within the same particle diameter range marked with different letters indicate significant differences at $p < 0.05$ (n=3).
